# Utilidad del test de TSH recombinante en el seguimiento de pacientes con cáncer diferenciado de tiroides según los resultados de tiroglobulina basal

**DOI:** 10.1515/almed-2020-0001

**Published:** 2020-03-19

**Authors:** Amaia Sandúa, Mónica Macías, Carolina Perdomo, Juan Carlos Galofre, Roser Ferrer, Estibaliz Alegre, Álvaro González

**Affiliations:** Servicio de Bioquímica, Clínica Universidad de Navarra, Avenida Pío XII 36, 31008 Pamplona, Spain; Departamento de Endocrinología, Clínica Universidad de Navarra, Pamplona, Spain; Instituto de Investigación Sanitaria de Navarra (IdiSNa), Pamplona, Spain; Hospital Universitario Vall d'Hebron, Barcelona, Spain

**Keywords:** anticuerpos antitiroglobulina, cncer de tiroides diferenciado, prueba de estimulación con rhTSH, tiroglobulina, tiroidectomía

## Abstract

**Introducción:**

La tiroglobulina (Tg) es el test de referencia en el seguimiento del cáncer diferenciado de tiroides (CTD). La detección de Tg se puede mejorar mediante el empleo de hormona estimulante de la tiroides (TSH) humana recombinante (rhTSH). El objeto del presente estudio es evaluar la utilidad de las pruebas de estimulación con rhTSH cuando se emplean tests de Tg de alta sensibilidad.

**Métodos:**

Se realizó un análisis retrospectivo de los resultados de 181 tests de rhTSH realizados a 114 pacientes con CTD con autoanticuerpos antitiroglobulina (anti-Tg) negativos. Se realizaron estudios de imagen a todos los pacientes. Los niveles de Tg y anti-Tg se midieron mediante inmunoensayos específicos.

**Resultados:**

La estimulación de RhTSH en pacientes con concentraciones basales de Tg (b-Tg) inferiores a 0.2 ng/mL siempre resultó en concentraciones de Tg estimulada por rhTSH (s-Tg) inferiores a 1.0 ng/mL y sin enfermedad estructural. De los 30 pacientes que presentaron concentraciones de b-Tg entre 0.2 y 1.0 ng/mL, solo un paciente mostró valores de s-Tg indicativos de respuesta bioquímica incompleta. Los pacientes con estudios de imagen negativos presentaron menores concentraciones de s-Tg, que aquellos con hallazgos inespecíficos o anormales (p < 0.05). El análisis de curvas ROC de s-Tg para la detección de alteraciones en los estudios de imagen arrojó un área bajo la curva (AUC) de 0.763 (p < 0.05). Con un punto de corte de s-Tg de 0.85 ng/mL, la sensibilidad fue del 100%, descendiendo al 96.15% cuando el punto de corte de s-Tg se estableció en 2 ng/mL.

**Conclusiones:**

El test de estimulación con rhTSH es útil para los pacientes con CTD con niveles de b-Tg iguales o superiores a 0.2 ng/mL.

## Introducción

Aunque el cáncer de tiroides diferenciado (CTD) representa menos del 1% de las neoplasias malignas, es el cáncer endocrino más prevalente, con una incidencia creciente en todo el mundo y mayor prevalencia entre las mujeres de mediana edad [1]. La mayoría de los pacientes con CTD presentan una histología papilar (95%), asociada a un mejor pronóstico. Entre las opciones terapéuticas, seleccionadas de acuerdo a una evaluación preoperatoria de riesgo [2], se encuentran la cirugía (lobectomía o tiroidectomía total), la ablación con yodo radiactivo (RAI), y la terapia supresora de la hormona estimulante de la tiroides (TSH) [2], [3]. Aunque el CTD tiene muy buen pronóstico, requiere un seguimiento activo [1].

La tiroglobulina (Tg) es una proteína sintetizada exclusivamente por lo tirocitos y producida tanto por células sanas como por tumorales [1], [3]. La ablación total del tejido tiroideo provoca una reducción de los niveles de Tg sérica hasta niveles indetectables. Por esta razón, la Tg es el biomarcador de referencia para el seguimiento del CTD [2], [4] y un factor clave en la estratificación dinámica de la respuesta al tratamiento [5]. De hecho, las recomendaciones de la American Thyroid Association (ATA) publicadas en 2015 clasifican la respuesta del paciente basándose en los niveles séricos de Tg basal (b-Tg) y estimulada (s-Tg) [2]. Sin embargo, existen dificultades técnicas en la cuantificación de la Tg, debido a que los diferentes ensayos tienen distinta sensibilidad [6], [7], así como a posibles interferencias, provocadas especialmente por la presencia de anticuerpos anti-Tg (anti-Tg) [8], [9], [10].

Los niveles de Tg circulante están relacionados con la carga tumoral; por lo tanto, es esencial aumentar la sensibilidad de las técnicas empleadas para su cuantificación, con el fin de lograr la detección temprana de enfermedad persistente o recurrente. Dado que la TSH estimula la producción de Tg, los niveles séricos de Tg se pueden incrementar mediante la inducción de hipotiroidismo, con la retirada de la terapia hormonal sustitutiva o con la administración de TSH humana recombinante (rhTSH) [11]. La estimulación con rhTSH (Thyrogen^®^) es una alternativa a la retirada de la tiroxina que previene las morbilidades que el hipotiroidismo ocasiona [12].

La estimulación conrhTSH tiene un coste elevado, precisando de la extracción de sangre en tres días distintos durante un periodo de 5 días. Esta prueba resultaba de gran utilidad con los antiguos métodos de cuantificación de Tg, que tenían una baja sensibilidad de entre 0.5 y 1.0 ng/mL [10], [13]. Sin embargo, los métodos de cuantificación de Tg que se emplean actualmente tienen mayor sensibilidad funcional, detectando niveles inferiores a 0.2 ng/mL [9], lo que pone en cuestión la utilidad de las pruebas de estimulación de Tg con rhTSH para la clasificación de los pacientes [2], [14]. La estimulación con rhTSH se utiliza en múltiples contextos y existe un amplio contexto en el grupo europeo sobre su empleo en pacientes con un riesgo medio o alto de recurrencia [15]. Aunque está ampliamente aceptado que no es necesario realizar la prueba de estimulación con rhTSH en pacientes con b-Tg en el límite de detección [6], [14], existen pocos datos sobre su utilidad en pacientes con valores de b-Tg indicativos de respuesta bioquímica indeterminada o incompleta. El propósito del presente estudio es evaluar la utilidad de la prueba de estimulación con rhTSH, cuando se emplea una técnica de cuantificación de Tg de alta sensibilidad. Un objetivo secundario de este estudio es analizar si las pruebas de laboratorio pueden resultar de utilidad a la hora de identificar en qué tipo de pacientes la estimulación con rhTSH va a proporcionar información relevante.

## Materiales y métodos

### Pacientes

Se realizó una revisión retrospectiva de 181 pruebas de estimulación con rhTSH realizadas durante el seguimiento de 114 pacientes con CTD (edad media = 45.7 ± 14.3 años; 73% mujeres). Un total de 102 pacientes presentaban cáncer papilar, mientras que los otros 12 restantes tenían cáncer folicular. Los criterios de inclusión fueron pacientes con CTD que se habían sometido previamente a una tiroidectomía total y habían recibido terapia RAI. Los pacientes con anticuerpos anti-Tg basales positivos se excluyeron para evitar imprecisiones en la cuantificación sérica de Tg. El estadiaje del CTD se realizó aplicando los criterios de la 8^a^ edición de la American Joint Committee on Cancer and Union Internationale Contre le Cancer (AJCC–UICC) [16]. El estudio recibió la correspondiente aprobación del Comité de Ética Institucional.

### Inmunoensayo

La TSH sérica se cuantificó mediante un inmunoensayo de electroquimioluminiscencia, realizado en un autoanalizador Cobas 8000 (Roche Diagnostics, Mannheim, Alemania). El rango de referencia indicador por el fabricante era 0.27–4.20 mIU/L, con una sensibilidad funcional de 0.005 mIU/L.

La Tg se midió mediante inmunoensayo de quimioluminiscencia en un autoanalizador Access II (Beckman Coulter, Nyon, Suiza). Este método ha sido estandarizado empleando el material de referencia CRM 457. De acuerdo con el fabricante, sus límites de detección y sensibilidad funcional son 0.01 ng/mL y 0.1 ng/mL, respectivamente [17].

Los anticuerpos anti-Tg se cuantificaron mediante inmunoensayo de quimioluminiscencia en el mismo equipo Access II (Beckman Coulter). Una muestra se consideró positiva si se detectaban anticuerpos anti-Tg (limite de detección: 0.9 IU/mL).

Todos los inmunoensayos fueron realizados en el mismo laboratorio, empleando los mismos métodos a lo largo de todo el estudio.

### Estimulación con rhTSH

Las pruebas de estimulación con rhTSH se realizaron con inyecciones de rhTSH (Thyrogen^®^, Genzyme, Cambridge, MA, EE.UU) [18]. De forma breve, tras la extracción de sangre para la cuantificación de b-Tg, anticuerpos anti-Tg y TSH, se inyectó Thyrogen^®^ (0.9 mg) por vía intramuscular dos días seguidos. Para comprobar que la estimulación era la adecuada, se cuantificaban los niveles de TSH al tercer día. Al quinto día de estimulación, se volvían a medir los niveles de s-Tg y de anticuerpos anti-Tg.

### Definición de la respuesta al tratamiento

El nivel de respuesta al tratamiento inicial se determinó conforme a los criterios para el cáncer de tiroides de ATA (2015) [2], esto es: a) la respuesta es “excelente” si los niveles de b-Tg son < 0.2 ng/mL, o s-Tg <1.0 ng/mL; b) “indeterminada” si los niveles de b-Tg son ≥0.2 y <1.0 ng/mL, o si los valores de s-Tg se encuentran entre ≥1 y <10 ng/ml; y c) “bioquímicamente incompleta” cuando los valores de b-Tg son ≥1.0 ng/mL o los valores de s-Tg son ≥10 ng/mL.

Según los niveles de b-Tg, se clasificó a los pacientes en tres grupos: Grupo A si Tg <0.2 ng/mL (respuesta excelente); Grupo B si 0.2 ≤ Tg < 1.0 ng/mL (respuesta indeterminada), y Grupo C si Tg ≥1.0 ng/mL (respuesta incompleta).

### Estudios de imagen

Todas las pruebas de estimulación con rhTSH iban acompañadas de estudios de imagen. Se realizó una ecografía a todos los pacientes que, en ocasiones, a criterio del endocrinólogo, iba acompañada de una tomografía computarizada, una resonancia magnética o una imagen nuclear funcional para identificar posibles metástasis locales o distantes.

### Análisis estadísticos

El análisis estadístico de los resultados se realizó con el programa GraphPad Prism versión 6.07 (La Jolla, CA, EE.UU). La distribución no-gaussiana de los datos se comprobó con el test de normalidad de Kolmogorov–Smirnov. Todos los datos están expresados como mediana y rango intercuartílico. La comparación de datos entre grupos se realizó mediante el test de Kruskal Wallis, seguido del test de comparaciones múltiples de Dunn. Las correlaciones se evaluaron con el coeficiente de correlación de Spearman. Las relaciones entre grupos se analizaron mediante la prueba *X* ^*2*^. Se construyeron curvas ROC para determinar el punto de corte para obtener la máxima sensibilidad. El nivel de concordancia se evaluó con el índice kappa de Cohen, que se interpretó de acuerdo con el siguiente criterio: 0.81–1,00 “muy bien”; 0.61–0.80 “bien”; 0.41–0.60 “moderado”; 0.21–0.40 “bajo” [19]. Un valor p bilateral de <0.05 se consideró estadísticamente significativo.

## Resultados

### Tiroglobulina basal y estimulada con rhTSH

Conforme a los criterios de ATA [2], nos basamos en los valores de b-Tg para la clasificación de los pacientes (Tabla 1): Grupo A (76 casos, 73 pacientes); Grupo B (70 casos, 30 pacientes), y Grupo C (35 casos, 20 pacientes). La mediana de concentración de TSH sérica al tercer día de estimulación fue 142 mIU/L (Q1–Q3: 115–185 mIU/L). Tal como se esperaba, no se encontró relación entre la TSH y la b-Tg o la s-Tg.

**Tabla 1:** Concentraciones de tiroglobulina basales y tras 5 días de estimulación con rhTSH.

Grupo	Tg basal (Respuesta bioquímica según los criterios ATA 2015 [2])	Pacientes, n	Casos, n	Mediana de Tg basal (rango intercuartílico)	Mediana de Tg tras estimulación rhTSH (rango intercuartílico)	Incremento en múltiplos respecto al basal (rango intercuartílico)
A	<0.2 ng/mL (Excelente)	73	76	0.1 ng/mL (sensibilidad funcional)	0.1 ng/mL (0.1–0.2 ng/mL)	
B	≥0.2 y <1.0 ng/mL (Indeterminada)	30	70	0.40 ng/mL (0.37–0.60 ng/mL)	3.9 ng/mL (1.8–5.3 ng/mL)	6.9 (2.9–11.4)
C	≥1.0 ng/mL (Incompleta)	20	35	2.6 ng/mL (1.48–3.63 ng/mL)	12.4 ng/mL (6.6–25.8 ng/mL)	4.29 (2.73–5.85)

**Figura 1:**
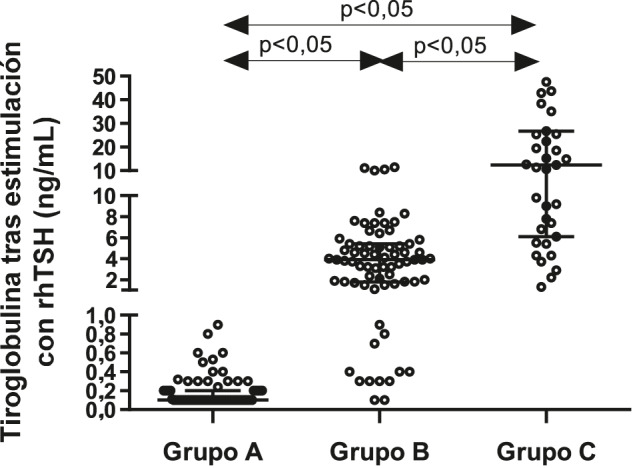
Niveles de tiroglobulina (Tg) tras estimulación con rhTSH de acuerdo a las concentraciones basales de tiroglobulina. Los pacientes fueron clasificados en función de sus niveles de Tg basal en Grupo A si Tg < 0.2 ng/mL; Grupo B si 0.2 ≤ Tg < 1.0 ng/mL, y Grupo C si Tg ≥ 1.0 ng/mL.

La estimulación con rhTSH en el Grupo A siempre se asoció a niveles de s-Tg inferiores a 1.0 ng/mL (Tablas 1 y 2), lo que corresponde a una respuesta excelente según los criterios ATA de estratificación del riesgo dinámico. Los niveles de Tg no aumentaron en 48 de las 76 pruebas realizadas, mientras que los niveles de s-Tg aumentaron en las restantes 28. Sin embargo, solo seis pacientes mostraron niveles entre 0.5 y 0.9 ng/mL (Figura 1).

Entre los pacientes con una respuesta indeterminada (Grupo B), siete presentaron incrementos inferiores al 100%, la totalidad de los cuales presentaron valores de b-Tg inferiores a 0.4 ng/mL. Se halló una correlación entre b-Tg y s-Tg (r = 0.394; p < 0.01). Los valores de s-Tg en el grupo de respuesta indeterminada fueron estadísticamente superiores a los observados en el grupo con una respuesta excelente (p < 0.05; Figura 1). En 54 casos (76%) los valores de s-Tg estuvieron asociados a una respuesta bioquímica indeterminada (s-Tg ≥ 1 y <10 ng/mL), mientras que en otros 12 casos (18.3%) los valores de s-Tg se asociaron a una respuesta excelente (s-Tg <1.0 ng/mL). Solo cuatro casos, todos ellos de un mismo paciente, resultaron en valores de s-Tg superiores a 10 ng/mL.

Finalmente, con respecto a los pacientes que mostraron una respuesta incompleta (Grupo C), no se observó una correlación estadísticamente significativa entre los niveles de b-Tg y s-Tg. En el Grupo C, los niveles de s-Tg fueron significativamente superiores a los de los otros grupo (p < 0.05; Figura 1). En 15 casos, los valores s-Tg resultaron en una respuesta indeterminada (s-Tg <10 ng/mL), mientras que en 20 casos, los valores de s-Tg indicaban una respuesta bioquímica incompleta. En tres casos correspondientes a tres pacientes distintos, los niveles de b-Tg fueron >10 ng/mL. Tal como se esperaba, los correspondientes niveles de s-Tg también fueron >10 ng/mL.

Considerando a la totalidad de los pacientes, se observó un alto nivel de concordancia entre la clasificación de la respuesta bioquímica basada en los valores de b-Tg y la realizada basada en s-Tg (índice kappa = 0.732; IC. 95% = 0.645–0,818). Sin embargo, al excluir a los pacientes con b-Tg <0.2 ng/mL (Grupo A), el nivel de concordancia bTg/s-Tg descendía a moderado (índice kappa = 0.392; IC 95% = 0.213–0.572).

### Tiroglobulina basal y estimulada por rhTSH en relación con los estudios de imagen

La totalidad de los estudios de imagen fueron negativos cuando los valores de b-Tg eran inferiores a 0.2 ng/mL (Grupo A, Tabla 2). En el Grupo B, las imágenes mostraron una respuesta indeterminada en el 24% (17) de los pacientes, y enfermedad persistente en el 13% (9), mientras que los estudios de imagen fueron negativos en el 63% (44) restante. Por último, en el Grupo C los estudios de imagen fueron negativos en el 26% de los casos (9), mostraron respuesta indeterminada en el 37% (13), y una respuesta estructural incompleta en el restante 37% (13). En los pacientes que presentaron valores de b-Tg >10 ng/mL, los estudios de imagen mostraron una respuesta estructural incompleta. El nivel de concordancia entre los estudios de imagen y los valores de b-Tg fue bajo (índice kappa = 0.324; I.C. 95% = 0.207–0.441).

**Tabla 2:** Concentraciones de tiroglobulina y estudios de imagen según la respuesta al tratamiento (criterios ATA 2015 [2]).

	Tiroglobulina estimulada con rhTSH			Imagen		
	Respuesta excelente	Respuesta indeterminada	Respuesta bioquímica incompleta	Negativa	Indeterminada	Enfermedad estructural
*Tiroglobulina basal*						
Respuesta excelente	76	0	0	76	0	0
Respuesta indeterminada	12	54	4	44	17	9
Respuesta bioquímica incompleta	0	15	20	9	13	13
*Imagen*						
Negativa	87	35	7			
Indeterminata	1	23	6			
Enfermedad estructural	0	11	11			

En el Grupo B, cuando los valores de s-Tg indicaban una respuesta excelente (12 casos), todos los estudios de imagen fueron negativos. Cuando los valores de s-Tg indicaban una respuesta indeterminada (54 casos), los estudios de imagen resultaron negativos en el 59%, mostraron una respuesta indeterminada en el 28%, y enfermedad persistente en el 13%. Del mismo modo, cuando los valores de s-Tg indicaban una respuesta bioquímica incompleta (4 casos), las imágenes resultaron negativas o indicaron una respuesta indeterminada en el 50% de los pacientes, y enfermedad persistente en el 50%.

En el Grupo C, cuando los valores de s-Tg indicaban una respuesta indeterminada (15 casos), los estudios de imagen fueron negativos o indeterminados en el 73% de los casos e indicaban enfermedad persistente en el 27% restante, mientras que si los valores de s-Tg correspondían a una respuesta bioquímica incompleta (20 casos), las imágenes resultaron ser negativas o mostraron una respuesta indeterminada en el 55% de los casos, y enfermedad persistente en el 45%.

**Figura 2:**
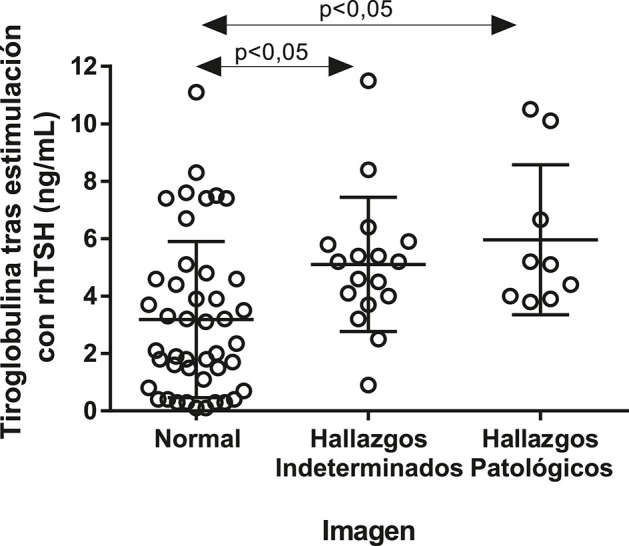
Niveles de tiroglobulina (Tg) tras estimulación con rhTSH de acuerdo a los hallazgos de imagen encontrados en el grupo de casos con tiroglobulina basal entre ≥ 0.2 ng/mL y < 1.0 ng/mL.

**Figura 3:**
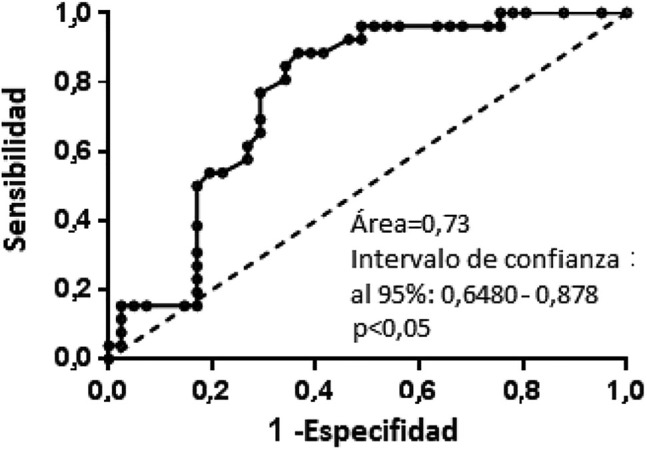
Análisis de la curva ROC de tiroglobulina estimulada con rhTSH para hallazgos de imagen anormales en los casos con tiroglobulina basal entre ≥ 0.2 ng/mL y < 1.0 ng/mL.

En el Grupo B, no se observaron diferencias basándonos en los estudios de imagen (datos no mostrados). Sin embargo, los pacientes con estudios de imagen negativos presentaron niveles de s-Tg significativamente inferiores (mediana de s-Tg = 2.2 ng/mL; Q1–Q3 = 0.9–4.6 ng/mL) a los que presentaron respuesta indeterminada (mediana de s-Tg = 5.2 ng/mL; Q1–Q3 = 3.2–5.9 ng/mL; p < 0.05) o resultados anormales (mediana de s-Tg = 5.1 ng/mL; Q1–Q3 = 3.9–8.4 ng/mL; p < 0.05) (Figura 2). Únicamente un paciente con enfermedad estructuralmente persistente según los estudios de imagen mostró valores de s-Tg superiores a 10 ng/mL. En el Grupo B, el análisis ROC de valores de s-Tg realizado para identificar alteraciones en los estudios de imagen arrojó una AUC de 0.763 (IC 95% = 0.6482–0.8780; p < 0.05) (Figura 3). Con un punto de corte de s-Tg de 0.85 ng/mL, la sensibilidad para detectar alteraciones en los estudios de imagen fue del 100% (ningún falso negativo), pero con una especificidad del 24%. Con un valor límite de s-Tg de 2 ng/mL, la sensibilidad descendió al 96.15% con una especificidad del 46%.

## Discusión

La prueba de estimulación con rhTSH se realiza en pacientes con CTD sometidos a una tiroidectomía más RAI para identificar enfermedad persistente o recurrente, o para reclasificar el riesgo de recurrencia [3]. Nuestros resultados muestran que, cuando se emplean tests de alta sensibilidad para Tg, si los valores de b-Tg se encuentran por debajo del límite de sensibilidad, los valores de s-Tg siempre serán inferiores a 1.0 ng/mL, lo cual también es indicativo de una respuesta excelente [2], [14], [20], y hace que sea innecesario realizar la prueba de estimulación de Tg [21], [22]. Pacini et al. observaron que los valores de b-Tg que quedaban por debajo del límite de detección, o s-Tg mínimamente detectables junto con la ausencia de anticuerpos anti-Tg identificaban con gran fiabilidad a los pacientes libres de enfermedad [23]. Además, los estudios de imagen de todos estos pacientes resultaron negativos, mostrando una respuesta excelente al tratamiento. En coherencia con estos resultados, Verburg et al. [24] publicaron recientemente que los niveles bajos o indetectables de b-Tg hacen innecesarias las ecografías del cuello. Si b-Tg ≥1.0 ng/mL, la estimulación con rhTSH no parece aportar información adicional a la obtenida mediante el sistema de estratificación del riesgo basado en la evidencia bioquímica [2]. Sin embargo, sería necesario investigar si los niveles de s-Tg son un factor predictivo de metástasis local o distante en pacientes con valores b-Tg ≥1.0 ng/mL.

El Grupo B de pacientes se considera una zona indefinida donde las pruebas de Tg estimulada por rhTSH puede aportar información relevante [6]. Algunos autores han encontrado enfermedad recurrente en el 12.5% de los pacientes con bajo riesgo y concentraciones de b-Tg >0.15 ng/mL [25]. En algunos pacientes con valores de b-Tg entre 0.2 y 1.0 ng/mL, las concentraciones de s-Tg pueden llevar a reclasificar la respuesta como respuesta bioquímica incompleta, lo cual tiene implicaciones terapéuticas, como un seguimiento más estrecho mediante la realización de estudios de imagen o la realización de nuevos procedimientos terapéuticos. Los estudios de imagen de todos los pacientes con concentraciones de s-Tg <0.85 ng/mL fueron negativos, sin mostrar evidencia de enfermedad estructural. Este hallazgo concuerda con los resultados de otros estudios, donde las concentraciones de s-Tg <0.5 ng/mL indicaron una probabilidad del 98% de estar libre de enfermedad [13].

Los valores de b-Tg y s-Tg solo mostraron correlación en el Grupo B, lo que contrasta con estudios anteriores, donde se ha observado una correlación entre b-Tg y s-Tg en un amplio rango de valores de b-Tg (0.05 a 1,000 ng/mL) [11]. Estas diferencias podrían ser debidas a diferencias metodológicas, a la variedad en el tipo de pacientes y el momento de cuantificación de s-Tg, ya que estos autores la cuantificaban a las 72 h de la estimulación.

Todas estas conclusiones se han obtenido bajo la premisa de que todos los pacientes eran anticuerpos anti-Tg negativos. Es sabido que la presencia de anticuerpos anti-Tg puede causar interferencias en los inmunoensayos, ya que provocan un descenso en las concentraciones de Tg [6], [26]. Sin embargo, un resultado negativo para la presencia de anti-Tg no significa que no pueda haberse producido una posible interferencia, ya que la prueba puede no haber detectado la presencia de anti-Tg [8], [9]. También se debe tener en cuenta que puede haber un tumor agresivo poco diferenciado con una síntesis limitada de Tg que haga que el análisis sea negativo o muestre valores bajos de b-Tg [6].

**Figura 4:**
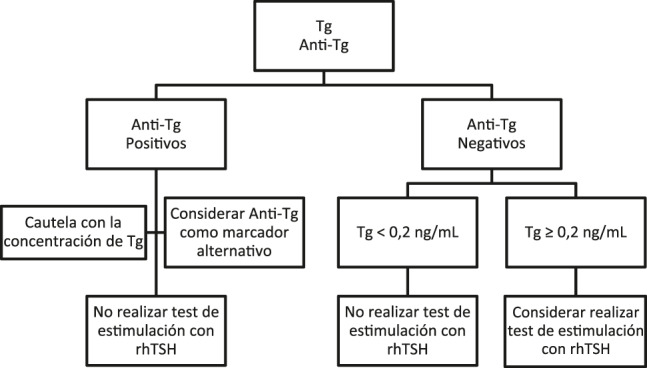
Propuesta de diagrama de flujo para la selección desde el laboratorio de pacientes que podrían beneficiarse de la prueba de estimulación con rhTSH.

Desde el punto de vista analítico, proponemos el siguiente protocolo, en caso de que plantee una determinación de Tg estimulada por rhTSH (Figura 4): En primer lugar, realizar un test de b-Tg empleando una técnica con una sensibilidad funcional de 0.2 ng/mL o inferior [6] y una prueba de anti-Tg. Si la muestra es positiva para anti-Tg, considere la posibilidad de no realizar el test de estimulación, ya que la cuantificación de Tg puede arrojar resultados imprecisos o poco fiables debido a posibles interferencias [4]. En estos casos, los anticuerpos anti-Tg pueden considerarse un biomarcador subrogado. Si la muestra es negativa para anti-Tg y b-Tg <0.2 ng/mL, no realizar la prueba de estimulación con rhTSH, ya que ésta no aportaría información clínica adicional. Si b-Tg ≥0.2 ng/mL, se puede realizar un test de estimulación con rhTSH. Giovanella et al. propusieron un diagrama de flujo similar [9], aunque la cuantificación de anti-Tg no se incluyó, la cual es para nosotros esencial a la hora de evitar posibles interferencias. Siguiendo este sencillo protocolo, el número de pruebas de estimulación con rhTSH se limitaría significativamente a aquellos casos en los que aporte un beneficio, reduciendo así costes y evitando la realización de pruebas innecesarias a los pacientes.

En resumen, la prueba de estimulación con rhTSH no resulta de utilidad en algunos pacientes con CTD y concentraciones de b-Tg inferiores a 0.2 ng/mL, mientras que sí puede serlo en otros pacientes. El papel del laboratorio en la identificación de estos pacientes es crucial, ya que proporciona resultados iniciales. Por lo tanto, la comunicación laboratorio-especialista es esencial para la implementación del algoritmo propuesto.
